# Malignant Melanoma of the Urethra: A Rare Histologic Subdivision of Vulvar Cancer with a Poor Prognosis

**DOI:** 10.1155/2012/385175

**Published:** 2012-12-20

**Authors:** Veronika Günther, I. Alkatout, C. Lez, S. Altarac, R. Fures, H. Cupic, Z. Persec, Z. Hrgovic, C. Mundhenke

**Affiliations:** ^1^Department of Gynaecology and Obstetrics, University of Kiel, 24105 Kiel, Germany; ^2^Department of Pathology and Cytology, General Hospital Zabok, 49210 Zabok, Croatia; ^3^Department of Urology, General Hospital Zabok, 49210 Zabok, Croatia; ^4^Department of Obstetrics and Gynaecology, General Hospital Zabok, 49201 Zabok, Croatia; ^5^Ljudevit Jurak Department of Pathology, Sestre milosrdnice University Hospital, HR-10000 Zagreb, Croatia; ^6^Department of Urology, University Hospital Dubrava, HR-10000 Zagreb, Croatia; ^7^Department of Gynaecology and Obstetrics, Johann Wolfgang Goethe University, 60590 Frankfurt, Germany

## Abstract

Malignant melanoma of the urethra is a rare tumour that is difficult to diagnose and treat, resulting in a poor prognosis. In this paper, we present the case of a 65-year-old woman who was referred to a gynaecologist because of a urethral mass that mimicked a caruncle. The tumour was removed by local excision, and a pathological analysis revealed a malignant melanoma. Distal urethrectomy was performed after three months with no evidence of residual tumour. There was no evidence of disease at a six-year followup. In this paper, we compare the epidemiology, treatment, staging, and prognosis of vulvar cancer in general to malignant melanoma of the vulva in particular.

## 1. Introduction

Vulvar cancer can be divided into several histological types. The most common type is squamous cell carcinoma (90%), whereas melanoma comprises only 2 to 9% of malignant tumours of the vulva. This incidence notwithstanding, melanoma represents the most common type of vulvar cancer. The most common origin sites for primary malignant melanoma are the labia minora, followed by the labia majora and the area around the clitoris [1]. Malignant melanoma has five main histologic subtypes: (1) superficial spreading melanoma; (2) nodular melanoma, which has the worst prognosis; (3) lentigo maligna melanoma; (4) acral lentiginous melanoma; (5) desmoplastic melanoma [1].

Even with major surgery, radiotherapy, or immunotherapy, malignant melanoma of the urogenital tract generally has a poor prognosis [2]. Because of its nonspecific symptoms and localisation, malignant melanoma of the vulva is often diagnosed in more advanced stages. Staging is based on the Chung-Level and predicts survival. Survival ranges from long-term survival for levels I and II, 40% five-year survival for levels III and IV and 20% five-year survival for level V [3].

In the following paper, we present the case of a patient who is alive and free of recurrence six years after the initial diagnosis of malignant melanoma of the vulva. 

## 2. Case Report

**Figure 1:**
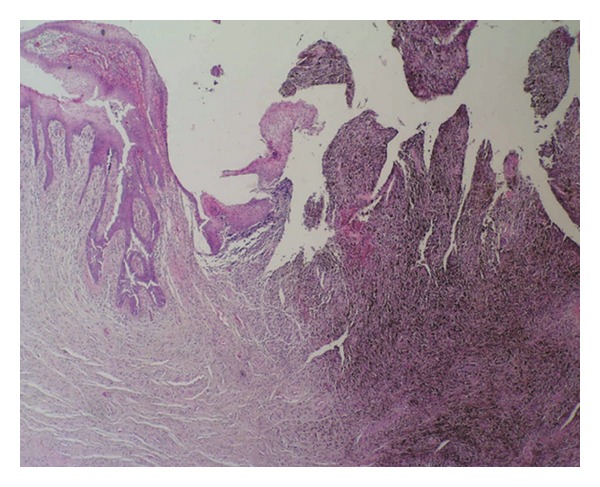
Polypoid, partially ulcerated melanoma of the female urethra (haematoxylin and eosin stain ×40).

**Figure 2:**
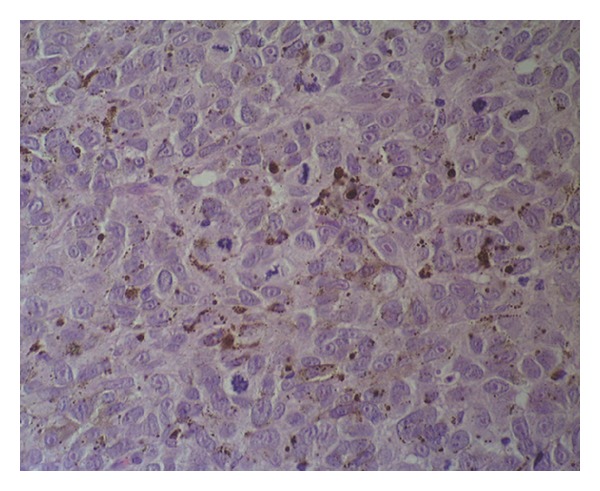
Nests of atypical melanocytes, with the large nuclei showing prominent nucleoli and numerous mitotic figures (haematoxylin and eosin stain ×400).

**Figure 3:**
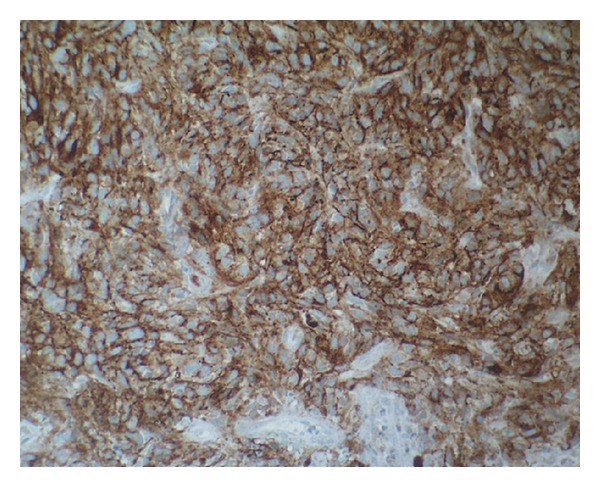
HMB-45 expression in melanoma cells (the immunohistochemical stain HMB-45, MSIP ×200).

A 65-year-old woman was referred to a gynaecologist for a urethral mass that on gross examination appeared to be a caruncle. Upon examination, a 3-cm wide, pedunculated, black pigmented, friable, haemorrhagic polyp was found at the posterior wall of the urethral meatus. The tumour was removed by local excision. A histopathologic analysis partially revealed a polypoid tumour with superficial ulceration that comprised squamous and transitional epithelia (Figure 1). The tumour was composed of loosely cohesive nests of atypical epithelioid and spindle-shaped melanocytes that showed diffuse and nested growth patterns. The neoplastic cells had an abundant eosinophilic cytoplasm, large hyperchromatic nuclei with prominent nucleoli, and brisk mitotic activity. Most of the tumour cells contained coarsely granular melanocytic pigment (Figure 2). The depth of tumour invasion, as measured by a digital microscopic camera (Olympus DP10), was 3.57 mm. No vascular/lymphatic invasion was observed histologically. Immunohistochemically, the tumour cells showed strong cytoplasmic reactivity for HMB-45 and S100 (Figure 3). Three months after the initial surgery, a resection of the distal third of the urethra showed no evidence of disseminated disease. The initial staging, which included computerised tomography (CT) of the chest, abdomen, and pelvis, revealed no evidence of disseminated disease. A repeat CT of the chest, abdomen, and pelvis two years after the initial diagnosis revealed two enlarged right parailiac lymph nodes, which remained unchanged at two subsequent CT assessments that were performed four and six years after the primary diagnosis. The patient showed no evidence of disease during a six year follow-up period. 

### 2.1. Epidemiology

It has been suggested that vulvar cancer exists as two separate diseases. The first type involves human papillomavirus (HPV) infection, which leads to vulvar intraepithelial neoplasia (VIN), a predisposing factor for vulvar cancer. An estimated 80% of untreated women suffering from VIN III develop invasive disease [6]. This type of vulvar cancer often develops in younger patients, and a recent review noted that approximately 15% of all vulvar cancers occur in women under age 40 [7]. Other predisposing factors, for example, a history of condylomata or sexually transmitted diseases (STD), low-economic status or nicotine abuse, have also been found [8].

The second type of vulvar cancer involves vulvar nonneoplastic epithelial disorders (VNED) and advanced age, which lead to cellular atypia and eventually to cancer [9]. 

Malignant melanoma of the vulva is a tumour of elderly women 65 to 75 [8]. Other factors, such as parity, genetic factors, or hormonal influences, do not appear to be related to either the occurrence or extent of vulvar melanoma. The relationship between ultraviolet radiation exposure and the risk for malignant melanoma has been known for some time; however, the knowledge of this correlation has not helped to clarify the aetiology underlying vulvar melanoma. It has been posited that UV light may be directly involved in the pathogenesis of this condition by causing a cell-mediated, systemic alteration of the immune response, which then increases the risk for vulvar melanoma [10]. Melanoma is a tumour originating from the neuroectoderm. Vulvar melanoma may develop from preexisting junctional or compound nevi as well as de novo from the melanocytes resting in the basal layer of squamous epithelium [11].

### 2.2. Clinical Features

The most frequently reported symptom of vulvar cancer is a long history of pruritus. Less commonly presenting symptoms include vulvar bleeding, discharge, dysuria, and pain. The most obvious presenting sign of vulvar cancer is a vulvar lump or mass, which may appear ulcerated, leukoplakic, fleshy, or warty.

In contrast, patients with malignant melanomas arising from the distal part of the urethra suffer from rapidly developing, nondistinct symptoms. These signs and symptoms include a urethral mass, nonspecific perineal pain, dysuria, incontinence, haematuria or local bleeding, and pruritus. The melanoma is usually pigmented and varies in colour from black to blue or light brownish; the lesions are firm, nodular, and often ulcerated. 

### 2.3. Staging

Vulvar cancer is staged using the TNM classification system. Staging reflects the characteristics of vulvar cancer growth, which occurs in the following manner: first by direct extension into the adjacent organs (the vagina, urethra, and anus), followed by lymphatic metastasis to the local lymph nodes (from the inguinal to the femoral to the pelvic lymph nodes) and finally by haematogenous spread to distant sites (liver, lungs, and bones) [4].

In malignant melanoma, there are several systems to describe the extent of lesions in addition to the TNM staging system.

1. The Clark-Level [12] delineates five levels of tumour invasion in cutaneous melanoma based on the penetration of dermal connective tissue planes and their correlation with prognosis, irrespective of the volume or the superficial tumour spread.
2. The Breslow-Index, levels I–IV, [13] uses tumour thickness as the most significant measurement of size and correlation with prognosis.
3. The Chung-Levels, levels I–V, [14] also describe the depth of tumour invasion and tumour thickness, while taking the lack of papillary dermis in the mucous membranes of the labia into account. This classification is often used to estimate the risk of regional and distant metastases [15].

Although it is primarily used for squamous cell carcinomas of the vulva, most investigators have found the FIGO classification to be of minimal prognostic value with respect to vulvar melanomas [16].

### 2.4. Treatment

In cases of suspicious vulvar lesions, even if the patient is asymptomatic, a biopsy must be performed down to the dermis for a histological diagnosis and to measure the tumour thickness. The affected area must be examined immunohistopathologically (Vimentin, S-100-Protein, HMB45) for further classification [17].

Other diagnoses that may present without any evidence of tumour cells, such as melanosis vulvae, melanocytic naevi, pigmented VIN, seborrhoeic keratosis, or angiokeratoma, should be excluded by biopsy as well. 

Surgical resection is the gold treatment standard in patients with vulvar cancer. Tumours <1 mm should be removed with a 1 cm margin, whereas radical vulvectomy is recommended if the tumour is >1 mm. Surgery should completely remove the cancer and identify the extent of disease to determine the stage of the lesion and the need for further therapy. The extent of disease determines the amount of surgery needed [18]. 

Radical vulvectomy with bilateral dissection of the inguinal and pelvic nodes was initially recommended as the standard treatment for most patients. The current recommendation is that a more individualised and conservative approach be used to treat such patients. Depending on the tumour's localisation, the extent of stromal invasion, and the general staging, it may be possible to choose radical local excision for treatment [4]. In cases of a malignant melanoma of the vulva located at the distal part of the urethra, treatment includes a partial or total urethrectomy (depending on the level of invasion) [19]. 

For vulvar cancer, the need for a lymphadenectomy depends on the stromal invasion. Stromal invasion of <1 mm is not associated with inguinal node metastases, whereas a patient with a tumour thickness >1 mm must be treated using inguinal-femoral lymphadenectomy, or at least sentinel lymph node biopsy in cases of inconspicuous groins [18]. If three or more inguinal-femoral lymph nodes are positive or if there is a macrometastasis (>10 mm), pelvic lymphadenectomy is recommended [18]. 

Because of the small number of cases, the treatment of vulvar malignant melanoma is similar to that of cutaneous malignant melanoma. Melanomas with <1 mm of dermal invasion must be removed with a 1 cm margin, whereas melanomas with >1 mm of dermal invasion should be surged with a 2-3 cm margin. In cases where there is adverse localisation (paraurethral), a radical vulvectomy with a partial urethrectomy and colpectomy should be discussed [20]. 

Sentinel lymph node biopsy (SLN) is also being recommended in selected patients who have early stage vulvar cancer to avoid the operative morbidity that is associated with inguinofemoral lymphadenectomy, such as wound complications or lower extremity oedema [21, 22]. Sentinel lymph node mapping was initially used to identify regional lymph node metastases in breast cancer and cutaneous melanoma and has now been evaluated in patients with early stage vulvar cancer, as well as malignant melanoma of the vulva with a tumour thickness <1 mm [23–25]. The sentinel lymph node can be detected using injected radio colloid 99mTc and isosulfan blue, which are preoperatively injected around the lesion. A hand-held gamma detection device is used to identify the sentinel lymph node(s) [26]. It is estimated that only 25–30% of patients with early stage vulvar cancer have lymph node metastases [21]. A patient with a positive sentinel node should undergo a full inguinofemoral lymphadenectomy followed by postoperative radiation therapy to the involved groin and pelvis. If the sentinel lymph nodes identified by mapping are histologically negative, no further treatment is indicated. The SLN may be used if (clinically and sonographically) there are no suspicious inguinal-femoral lymph nodes and if the tumour is not thicker than 1 mm (T1) [18]. Because of the rare incidence of malignant melanoma of the vulva, no SLN standards have been published; however, there are several studies that have shown the benefit of SLN, such as the smaller number of postoperative complications (e.g., lymphedema, deep venous thrombosis, and groin wound infections) [27].

Primary radiotherapy or radiochemotherapy, which is sometimes used as the sole treatment, is recommended for cases of more advanced disease in which surgical resection (with the aim of an R0-situation) is not possible. If there are more than three positive inguinal lymph nodes or if the vulvar cancer is not completely removed (R1 or <1 cm margin without the possibility of follow-up resection), adjuvant radiotherapy is sometimes recommended. Chemotherapy has poor response rates and is ineffective in treating recurrent disease after surgical resection [4, 17]. Similar to cutaneous melanoma, adjuvant therapy with interferon-alpha should be discussed for malignant melanoma of the vulva >1.5 mm. Interferon-alpha supports the immune system in eradicating solitary tumour cells after surgery and is associated with a longer disease-free survival time [28]. 

Those patients with a previously treated metastatic melanoma also benefit from ipilimumab, a new antibody therapy. This fully human monoclonal antibody binds to CTLA-4 (cytotoxic T lymphocyte-associated antigen 4), a molecule on cytotoxic T lymphocytes that is believed to play a critical role in regulating natural immune responses [29]. Ipilimumab is designed to block the activity of CTLA-4, thereby sustaining an active immune response to cancer cells [30].

### 2.5. Prognosis

The prognosis of patients with vulvar cancer is generally good when appropriate treatment is provided in a timely manner. The overall five-year survival rate (Table 1) is 70% and correlates with the disease stage and lymph node status. The number of positive inguinal lymph nodes is the most important prognostic factor [4].

In contrast to squamous cell carcinoma, there are limited reliable data concerning prognostic factors for nonsquamous cell vulvar malignancies. The thickness and depth of invasion of vulvar melanoma (as described by Breslow and Clark), as well as the regional lymph node metastasis, has been shown to correlate with the pattern of spread and prognosis of vulvar melanoma [15, 31]. Breslow's classification describes tumour thickness (mm), as measured by the distance between stratum granulosum and the deepest tumour cells [13]. 

The depth of invasion classified by Clark describes tumour invasion in relation to the layer of skin (e.g., tumour restricted to the epidermis = level I; infiltrating the stratum papillare = level II) [12].

Ulceration in melanomas presumably reflects very aggressive tumour growth that infiltrates and destroys the mucosal membrane. In general, vulvar melanoma carries a poor prognosis and has a tendency to recur locally as well as to develop distant metastases [32].

Vulvar melanoma has a local recurrence rate between 30 and 51%. The preferred site of recurrence is the groin, followed by the perineum, the rectum, the vagina, the urethra, and the cervix. In patients who develop a recurrence, the disease-free period averages one year and ranges from one month to 14 years [33].

## 3. Comment on the Case Report

Because malignant melanoma of the vulva is a rare tumour, surgeons should be mindful when examining suspicious lesions and be aware of the appropriate treatment options for the condition. In cases of suspicious lesions, a biopsy is recommended for the histological diagnosis and to determine appropriate treatment options, which are dependent on the tumour thickness. All of the patient's skin should be examined, and the tumour marker S100 should be measured after there is histological evidence of malignant melanoma. A chest X-ray, abdominal sonography, and sonography of the inguinal lymph nodes should be performed preoperatively. In cases of deep dermal invasion, examinations, such as CT of the thorax, CT of the abdomen, cystoscopy, and proctoscopy, should follow.

In the presented case, a lymphadenectomy was not performed. Based on the current standard, at minimum, a sentinel lymph node biopsy should be performed (if tumour thickness >1 mm) if there are inconspicuous inguinal lymph nodes. After the histological diagnosis, definitive surgical treatment should be initiated immediately.

## 4. Summary

Over the past decade, an increase in vulvar intraepithelial neoplasms and VIN-related invasive vulvar cancer has been noted in women under age 50. There is a statistically significant correlation between HPV infection, cigarette smoking, sexual transmitted diseases, and low-economic status and the incidence of vulvar cancer [34].

Compared with vulvar cancer in general, vulvar malignant melanoma is still a cancer of elderly women (65 to 75 years). In contrast to vulvar cancer, malignant melanoma is not VIN-associated. However, it has been discussed that UV light exposure over several decades may cause a cell-mediated systemic alteration of the immune response, thus predisposing patients to vulvar melanoma [10]. 

Because of the small number of cases [35], the treatment of vulvar malignant melanoma is similar to that of cutaneous malignant melanoma. Melanomas <1 mm thick must be removed with a 1-cm margin, whereas melanoma >1 mm thick should be surged with a 2-3-cm margin. 

An inguinal sonography of the lymph nodes should be performed preoperatively. In cases of inconspicuous nodes, a sentinel lymph node biopsy is recommended to prevent postoperative complications, such as lymphedema. In cases of suspicious nodes, a radical lymphadenectomy that includes the inguinal-femoral nodes should be conducted, whereas a pelvic lymphadenectomy has not been noted to confer additional benefits. 

The use of the sentinel lymph node biopsy in vulvar malignant melanoma cases is derived from the treatment guidelines for cutaneous malignant melanoma, which were developed by dermatologists. This approach has been efficacious in treating this condition and is currently the gold standard in lymph node surgery.

To guarantee optimal outcomes, a multidisciplinary approach that involves gynaecologists, pathologists, dermatologists, radiologists, and so forth should be implemented to treat malignant melanoma of the vulva.

**Established Facts**

- Vulvar cancer is related to VIN, HPV-infection, and low-economic status.
- Clinical symptoms of vulvar cancer: pruritus, bleeding, discharge, dysuria, and pain.
- Vulvar cancer is staged using the TNM classification system.

**Novel Insights**

- Vulvar malignant melanoma is a cancer of elderly women, not VIN-, but probably UV light exposure associated.
- Clinical symptoms of vulvar malignant melanoma: rapidly developing, nondistinct symptoms.
- The Clark-Level, Breslow-Index, and Chung-Level are used in addition to the TNM classification system.
- The treatment of vulvar malignant melanoma is similar to that of cutaneous malignant melanoma (e.g., an adjuvant therapy with interferon-alpha or a supporting antibody therapy with ipilimumab).

## Figures and Tables

**Table 1:** 

Clinical FIGO stage	Five-year survival (%)
I	98
II	85
III	74
IV	31
Inguinal-femoral lymph node status (all stages)	
Positive	52.4
Negative	91.3
Positive pelvic nodes	11

Information from [4, 5].
